# A Novel Sensitive Recombinase-Aided Amplification Integrated Test Strip for *Pseudomonas fluorescens* in Milk via Dual Gene Probes

**DOI:** 10.3390/bios15080553

**Published:** 2025-08-21

**Authors:** Guangying Zhang, Lili Zhang, Jingqin Ye, Dongshu Wang, Ying Lu

**Affiliations:** 1 College of Food Science and Technology, Shanghai Ocean University, Shanghai 201306, China; zzz_guangying@163.com (G.Z.); liliq1730@163.com (L.Z.); 2 Laboratory of Quality and Safety Risk Assessment for Aquatic Products on Storage and Preservation (Shanghai), Ministry of Agriculture, Shanghai 201306, China; 3 State Key Laboratory of Pathogen and Biosecurity, Beijing Institute of Biotechnology, Beijing 100071, China; yejq0922@163.com; 4 Marine Biomedical Science and Technology Innovation Platform of Lingang New Area, Shanghai 201306, China

**Keywords:** *Pseudomonas fluorescens*, recombinase-aided amplification, test strip, PMAxx, *aprX*, *gyrB*

## Abstract

*Pseudomonas fluorescens* is the main spoilage bacterium in milk, and its proliferation is one of the factors leading to the deterioration of the quality of raw milk. In this study, a rapid detection system for *P. fluorescens* was developed based on recombinase-aided amplification combined with a test strip (RAA-TS), which contained a double test line (DTL) targeting the virulence gene *aprX* of *P. fluorescens* and the housekeeping gene *gyrB* of *Pseudomonas*. Visual observation could detect *gyrB* (50 CFU/mL) and *aprX* (250 CFU/mL) within 90 min, including sample pretreatment and RAA reaction and detection steps. No cross-reactions were observed with *Pseudomonas* or other bacteria (n = 19). The quantitative detection limits (LOD) of *gyrB* and *aprX* for *P. fluorescens* in milk were 37 CFU/mL and 233 CFU/mL, respectively. Compared with polymerase chain reaction-agarose gel electrophoresis (PCR-AGE), the sensitivity of the developed RAA-TS-DTL system was increased by approximately four times. Furthermore, it could detect live *P. fluorescens* in milk when combined with optimized sample pretreatment by propidium monoazide (PMAxx). Its consistency with the traditional culture method in the detection of *P. fluorescens* spiked in milk samples (n = 25) was 100%. The developed RAA-TS-DTL had the advantages of high accuracy and short time consumption. Thus, it provides a new way or tool for the rapid screening or detection of *P. fluorescens* in milk.

## 1. Introduction

Milk is an ideal natural food since it contains an abundance of calcium, vitamin D, and essential amino acids, which are vital for human growth and development [1]. In 2022, the “Dietary Guidelines for Chinese Residents” increased the recommended daily milk intake to 300–500 g. To prevent spoiling, fresh milk requires strict cold-chain management and should be refrigerated at 2–6 °C [2]. Low temperatures could inhibit the activity of most microorganisms in raw milk [3]. However, it has been found that psychrophilic bacteria, including *Pseudomonas*, *Acinetobacter* and *Listeria monocytogenes*, can proliferate even in low-temperature environments [4]. They can rapidly dominate microbial populations, escalating from 50% to more than 90% in only a few days [5]. These *Pseudomonas* can produce extremely heat-resistant protease and lipase, making milk bitter, rotten, or clotted, resulting in spoilage [6]. Therefore, the rapid detection and control of *Pseudomonas* and other psychrophilic bacteria is key to ensuring the quality of fresh milk.

The current method for the detection of *Pseudomonas* encompasses microbial culture and an immunological and molecular biological technique. The microbial culture is widely used to detect *Pseudomonas*, but it is tedious and time-consuming. For example, Bouchair et al. [7] used a microbial culture method to identify the microbial populations in raw milk, taking more than 48 h. Enzyme-linked immunosorbent assay (ELISA) is the main immunological method for the detection of *Pseudomonas*. Matta et al. [8] developed a dot ELISA to detect *Pseudomonas* sp. AFT-36 in milk within 2.5 h with a detection limit of 1.01 ng/mL. Although it was highly accurate, it required specialized technical expertise and might have resulted in false-negative or false-positive results. Molecular biology techniques have been widely used to detect *Pseudomonas* due to the advantages of speed, sensitivity, and accuracy. For example, Xin et al. [9] developed a novel loop-mediated isothermal amplification (LAMP) method for detecting *Pseudomonas* in milk, achieving a detection limit of 48 CFU/reaction of template deoxyribonucleic acid (DNA). Yao et al. [10] developed a highly sensitive recombinase-aided amplification (RAA) method to detect *P. aeruginosa* in food; its detection time was merely 20 min, and its sensitivity was 1.7 pg/μL. RAA is a nucleic acid amplification technology that utilizes a recombinase, single-strand-binding protein, and DNA polymerase under isothermal conditions (generally 37–42 °C). In the presence of ATP, the recombinase first binds to the primer to form a protein–nucleic acid complex and then recognizes the template through homologous pairing. When the primer finds the matching DNA template, ATP hydrolysis provides energy, and the recombinase leaves the primer and synthesizes a new DNA strand under the action of DNA polymerase. Meanwhile, single-stranded DNA-binding protein (SSB) binds to the displaced single-stranded DNA to prevent the DNA template from re-forming a double strand. With the presence of energy and dNTP, the DNA polymerase completes the extension of the chain. This amplification process can be completed within 25 min under a constant temperature device [11]. Due to the excellent characteristics of RAA, such as high specificity and sensitivity, simple operation, and no need for specialized equipment [12], it can be used as an alternative to a polymerase chain reaction (PCR) or LAMP for developing on-site detection methods for pathogenic bacteria. Furthermore, Chen et al. [13] developed a centrifugal microfluidic point-of-care test by combining RAA with a CRISPR-Cas12a system, achieving the rapid detection of *P. aeruginosa* with a sensitivity of 10^3^ CFU/mL within 1.5 h. In addition, Yang et al. [14] used recombinase polymerase amplification combined with a lateral flow strip method to achieve the sensitive and specific detection of *P. aeruginosa*, and the consistency with the conventional culture method was 98.26%. Therefore, the current detection methods of *Pseudomonas* have the advantages of abundant species, simple operation, and high sensitivity.

*P. fluorescens*, a Gram-negative psychrophilic bacterium of the *Pseudomonas* genus [15], is renowned for its heat-resistant enzymes, making it a prevalent and detrimental psychrophile in the cold-chain logistics of milk [16]. In recent years, the techniques of ELISA, PCR, and LAMP have been used to detect *P. fluorescens*. For instance, Volk et al. [17] developed an ELISA method to detect *P. fluorescens* in milk, achieving a detection sensitivity of 21.0 ng/mL (AprX) for about 6–7 h. Min et al. [18] established a real-time PCR for *P. fluorescens* with a *gyrB* gene target, achieving a specific and sensitive detection (3.0 × 10^2^ CFU/mL). Furthermore, Maier et al. [19] developed a multiplex quantitative PCR (qPCR) method with a quantitative detection range of approximately 10^3^ to 10^7^ CFU/mL for target species, which could detect 262 CFU/mL of *P. fluorescens* for about 3 h. Additionally, Hu et al. [20] developed a real-time LAMP detection method for *aprX* and *gyrB* genes of *P. fluorescens*, achieving a detection limit of 2.2 × 10^2^ CFU/mL for 200 raw milk samples within 3 h, with a 100% concordance rate with the traditional culture methods (5–7 d). Although these methods are highly sensitive and accurate, they are time-consuming, and some methods require special equipment that does not meet the needs for the rapid field detection of *P. fluorescens* and early quality monitoring of milk. Furthermore, molecular biological methods cannot distinguish live and dead bacteria; thus, sometimes, there are false-positive or false-negative results [14].

**Figure 1:**
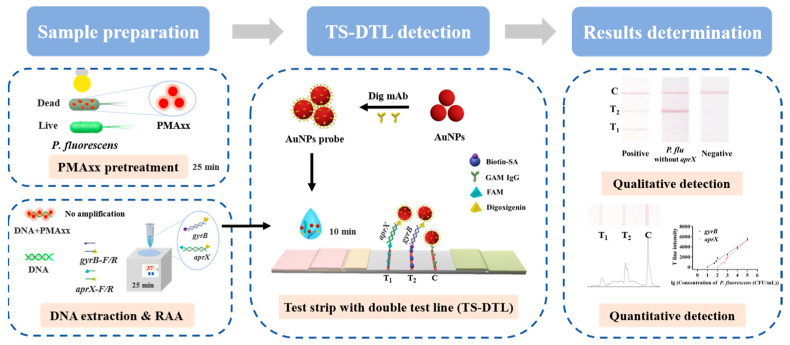
Schematic diagram for RAA-TS-DTL detection system for *P. fluorescens*.

PMAxx is a photoreactive dye with a high affinity to dsDNA, which cannot penetrate the cell membrane. Thus, when detecting cell populations containing dead and live bacteria, it can only selectively modify DNA exposed to incomplete cell membranes in dead cells, while DNA in live cells with intact cell membranes cannot be modified [21]. Thus, PMAxx can distinguish live and dead bacteria. Due to RAA, it is not only easy to operate and does not require special equipment, but it can also simultaneously complete the rapid amplification of multiple target genes within 25 min under constant temperature conditions, meeting the needs of on-site rapid detection. Thus, RAA was selected for the DNA amplification of live *P. fluorescens*. To develop a simple and accurate on-site detection method for live *P. fluorescens*, in this study, the virulence gene *aprX* of *P. fluorescens* and the housekeeping gene *gyrB* of *Pseudomonas* were employed as detection targets. Firstly, the 5′ end of the forward primer of the *aprX* and *gyrB* genes was separately labelled with FAM and biotin, and the 5′ end of the reverse primer of both genes was modified with digoxin. In order to capture two target genes, the test strip with the dual test line (TS-DTL) platform was constructed, where the T_1_ line was coated with a monoclonal antibody (mAb) against fluorescein isothiocyanate (FITC) to capture FAM-labelled *aprX* via an antigen-antibody interaction, and the T_2_ line was labelled with streptavidin to bind biotin-modified *gyrB* via a protein–ligand interaction. As shown in Figure 1, we optimized and established a sample pretreatment based on PMAxx to obtain the DNA of live *P. fluorescens*. Thus, the RAA products of *aprX* and *gyrB* were used as a sample for TS-DTL. AuNPs probe coated with the mAb against digoxin (Dig mAb-AuNPs) was employed to bind digoxin-modified *aprX* and *gyrB*, respectively; then, a sensitive RAA-TS-DTL system for *P. fluorescens* was achieved. Qualitative detection was performed by naked eye observation, while quantitative detection was carried out by Image J software to read the intensity value of the T line and the C line. Finally, detection performance, including specificity, sensitivity, accuracy, and so on, was evaluated. This study provides a new method and tool for the rapid screening and field detection of *P. fluorescens* in milk.

## 2. Materials and Methods

### 2.1. Materials and Reagents

Luria–Bertani broth (LB) was acquired from Land Bridge Technology Co., Ltd. (Beijing, China). A TIANamp Bacteria DNA Kit (containing lysis buffer, proteinase K, isopropanol, 70% ethanol, TE buffer, spin column, collection tube) was purchased from Tiangen Biotechnology Co., Ltd. (Beijing, China). The RAA basic reaction kit was purchased from Jiangsu Qitian Gene Biotechnology Co., Ltd. (Wuxi, China). Primers and template DNA were provided by Suzhou Genewiz Biotechnology Co., Ltd. (Suzhou, China). PMAxx was purchased from Shanghai Open Biotechnology Co., Ltd. (Shanghai, China). Ringer’s solution was obtained from Shanghai Zeye Biotechnology Co., Ltd. (Shanghai, China). A 2× PCR master Mix, 6× DNA loading buffer, SYBR Green Ⅰ nucleic acid fluorescent dye, agarose, etc., were purchased from Beijing Tsingke Biotech Co., Ltd. (Beijing, China). The fresh milk, UHT (Ultra-High Temperature) milk, cheese, yogurt, and milk powder were purchased from Lawson supermarket on campus. The frozen chicken breast, duck meat, pork belly, beef, Pacific white shrimp, Chinese mitten crabs, Pacific oyster, and Short Necked Clam were purchased from a local market in Shanghai.

### 2.2. Bacterial Sample Preparation and PMAxx Pretreatment

The bacterial strains used in this study are listed in Table S1. LB broth was used throughout to grow bacterial strains. All cultures were grown aerobically at 37 °C for 16 h with shaking. PMAxx can only selectively modify DNA exposed to incomplete cell membranes in dead cells. Based on this property of PMAxx dye, in this study, PMAxx was selected to pretreat the sample. According to the method of Huang et al. [22], 20 μM PMAxx was mixed with 1 mL of bacteria suspension and incubated in the dark for 10 min. Next, the mixture was exposed to a halogen lamp at a distance of 30 cm for 15 min.

The treated solution was used as a sample for DNA extraction. A TIANamp Bacteria DNA Kit was used to extract DNA. Briefly, 10 mL of dairy product samples were centrifuged at 3000× *g* for 6 min, and the obtained precipitate was incubated in 2 mL of lysis solution (Ligroin solution:0.5 M EDTA:TE Buffer = 10:3:2, *v*/*v*) for 1 min at room temperature. After centrifugation at 8000× *g* for 2 min, the obtained precipitate was mixed with 100 µL TE. Next, the mixture was lysed at 95 °C for 10 min, followed by an ice bath for 5 min. Finally, the lysate was centrifuged at 13,000× *g* for 3 min. The supernatant was stored at −20 °C for further use as DNA samples in RAA. The purity of the extracted DNA was assessed by determining the absorbance ratios at 260 nm and 280 nm (OD260/OD280). Furthermore, the molecular size, relative concentration, and integrity of the DNA fragments were ascertained through 1.5% agarose gel electrophoresis (AGE) analysis.

### 2.3. Primer Design and Screening for Target Genes

The complete CDS sequences of the *aprX* and *gyrB* genes were downloaded from NCBI (https://www.ncbi.nlm.nih.gov, accessed on 17 August 2025). Intraspecific (homology > 80%) and interspecific (homology < 30%) alignments of multiple genes were performed by DNAMAN software version 6.0. A total of 18 pairs of primers were designed by Primer Premier 5.0 (Premier Biosoft, Palo Alto, CA, USA). The amplification efficiency and specificity of primers were analyzed using NCBI-primer-BLAST.

After screening the primers, the 5′ end of the forward primer of the *aprX* and *gyrB* genes was separately labelled with FAM and biotin, and the 5′ end of the reverse primer of both genes was modified with digoxin, and then the extracted DNA was used for RAA amplification. According to the method of Christopher Maier [19], each pair of 10 μM synthesized primers was analyzed through a dissociation curve in qPCR to observe whether there was any non-specific amplification or primer dimer formation. The PCR of the primers was performed in a 25 μL reaction, containing 12.5 μL of a 2× PCR master mix, 0.5 μL of each 10 μM forward and reverse primers, 2 μL of template DNA, and 9.5 μL of ddH_2_O. The RAA of the primers was performed in an RAA reaction kit by incubation at 37 °C for 30 min. The obtained RAA products were analyzed by 2% AGE in a 1× TAE buffer at 100 V for 25 min.

### 2.4. Preparation of the AuNPs Probe

Red colloidal gold nanoparticles (AuNPs) were synthesized using the method of Lu et al. [23] with minor modifications. Briefly, 50 μL of a 10% HAuCl_4_ water solution (*w*/*v*) and 50 mL of ultrapure water were heated to boiling in a water bath. Subsequently, 2 mL of a 1% (*w*/*v*) Na_3_C_6_H_5_O_7_ aqueous solution was added and kept boiling for 15 min. When the solution color changed from colorless to wine-red and stabilized, the obtained AuNPs solution was cooled and stored at 4 °C. The absorption peaks of the AuNPs were measured by a UV-Visible spectrophotometer. A transmission electron microscope (TEM) and dynamic light scattering (DLS) were used to determine the size and morphology of AuNPs. Zeta potential was measured by a Zetasizer Nano ZS90 (Malvern, UK).

For the preparation of the AuNPs probe, 1 mL of an AuNPs solution (pH = 8) and 1 mg/mL of a Dig mAb solution were incubated at 25 °C for 2 h. Next, 50 μL of a 10% bovine serum albumin (BSA) water solution (*w*/*v*) was added and reacted for 30 min at 25 °C. The mixture was washed twice with a 500 μL PBST-BSA buffer (10 mM PBS containing 1% BSA, 5% sucrose, 0.5% Tween-20). After centrifugation at 5000× *g* for 20 min, the obtained precipitate was resuspended in a 100 μL PBST-BSA buffer containing 0.05% NaN_3_, which was used as an AuNPs probe solution and stored at 4 °C.

### 2.5. Test Strip Construction and Detection Procedure of RAA-TS-DTL

The RAA-amplified products, Dig mAb-AuNPs, and chromatographic buffer were mixed well to serve as samples for TS-DTL detection. For the preparation of TS-DTL, 2 mg/mL of FITC mAb and 0.5 mg/mL of streptavidin (SA) in PBS were sprayed onto a nitrocellulose (NC) membrane used as T_1_ and T_2_ lines, respectively. The C line was coated with 0.3 mg/mL GAM IgG. The NC membrane was dried at 37 °C for 2 h. TS-DTL was obtained by cutting the NC membrane into strips 5 mm in width, which were stored in aluminum foil bags at room temperature. For the detection of *P. fluorescens*, 10 µL RAA amplification products (5 µL *aprX* and 5 µL *gyrB*) were mixed with 10 µL of a chromatographic buffer (10 mM PBS containing 0.5% Tween-20, 20% BSA), 10 µL AuNPs-Dig probe, and 70 µL PBS. Subsequently, 100 µL of the mixture was added onto the sample pad of TS-DTL and left to stand for 15 min. For positive results, double T lines and a C line could be observed by the naked eye. If only the T_2_ and C lines were observed but the T_1_ line was absent, then the bacteria contained a *gyrB* gene but lacked an *aprX* gene. A negative result that was observed only on the C line indicated that *P. fluorescens* was not detected. The absence of the C line suggested that the test strip was invalid.

### 2.6. Optimization of the Reaction Conditions of RAA-TS-DTL

DNA extracted from *P. fluorescens* ATCC 13525 was used as the RAA amplification template to optimize the reaction temperature (20, 25, 30, 35, 37, 40, and 50 °C), time (5, 10, 15, 20, 25 and 30 min), and primer concentration (2.5, 5, 10, 20, and 40 μM). Furthermore, 16 µg of digoxigenin mAb with different AuNPs pH values (6.5, 7.0, 7.5, 8.0, 8.5, and 9.0) were used to prepare the AuNPs probes, and the absorbance values at 522 nm were determined. In addition, different concentrations of FITC mAb (1.0, 1.5, 2.0, and 2.5 mg/mL) in 10 mM PBS, and various amounts of digoxigenin mAb (8, 16, 24, and 32 µg), were chosen to optimize the detection conditions of RAA-TS-DTL. Tris-HCl (pH 6.8), PBS (pH 7.4), a carbonate-buffered saline solution (CBS, pH 9.6), a borate-buffered saline solution (BBS, pH 9.0), and a Tween borate-buffered saline solution (TBBS, pH 9.0) were individually used to optimize the chromatographic buffer of the RAA-TS-DTL.

### 2.7. Detection Performance of RAA-TS-DTL for P. fluorescens

#### 2.7.1. Specificity

The RAA amplification products of the *aprX* and *gyrB* from the 19 bacterial strains (Table S1) were used for specificity verification. Following the procedure in Section 2.5, 10 μL of the RAA amplification products were mixed with 10 µL of the chromatographic buffer, 10 µL of AuNPs-Dig probe, and 70 µL of PBS for testing.

#### 2.7.2. Sensitivity

A total of 10 µL of RAA amplification products from *P. fluorescens* with varying concentrations (from 10^1^ to 10^6^ CFU/mL) were mixed individually with 10 μL of a chromatography buffer, 10 µL of an AuNPs-Dig probe, and 70 μL of PBS. The mixture was added to the sample pad of TS-DTL. After 10–15 min, the presence of the C line and T line was observed, and images were taken. The intensity of the T lines was read by Image J software (U.S. National Institutes of Health, Bethesda, Maryland, USA, https://imagej.nih.gov/ij/, accessed on 17 August 2025). The qualitative sensitivity of RAA-TS-DTL was determined by the concentration of *P. fluorescens* when the colored T lines disappeared. The quantitative fitting curve was created by correlating the intensity of the T line with the concentration of *P. fluorescens*, and the quantitative sensitivity was calculated from the quantitative curve.

#### 2.7.3. Accuracy

One mL of different concentrations of *P. fluorescens* from 10^1^ to 10^6^ CFU/mL was mixed with 1 mL of UHT milk samples and detected by TS-DTL. The UHT-sterilized milk contaminated by *P. fluorescens* was detected as a microbial culture, RAA-TS-DTL, PCR-AGE, and qPCR, respectively.

#### 2.7.4. Stability

The storage stability of TS-DTL was assessed according to the method of Tang et al. [24], with slight modifications. The concentration of *P. fluorescens* was adjusted to 10^5^, 10^3^, and 10^1^ CFU/mL. Following the procedure in Section 2.2, the extracted DNA was stored at −20 °C for future utilization. TS-DTL without a sample addition was kept at 60 °C for 5 d. RAA products of different concentrations of *P. fluorescens* were daily detected according to the procedure in Section 2.5.

### 2.8. Testing of Spiked Food Samples

Three types of products, including dairy (n = 25), meat (n = 15), and aquatic products (n = 10), were used as spiked food samples. These samples were detected by the microbial culture method of China (SN/T 4044-2014), PCR-AGE, and RAA-TS-DTL, respectively.

## 3. Results and Discussion

### 3.1. Primer Screening of gyrB and aprX Genes

**Figure 2:**
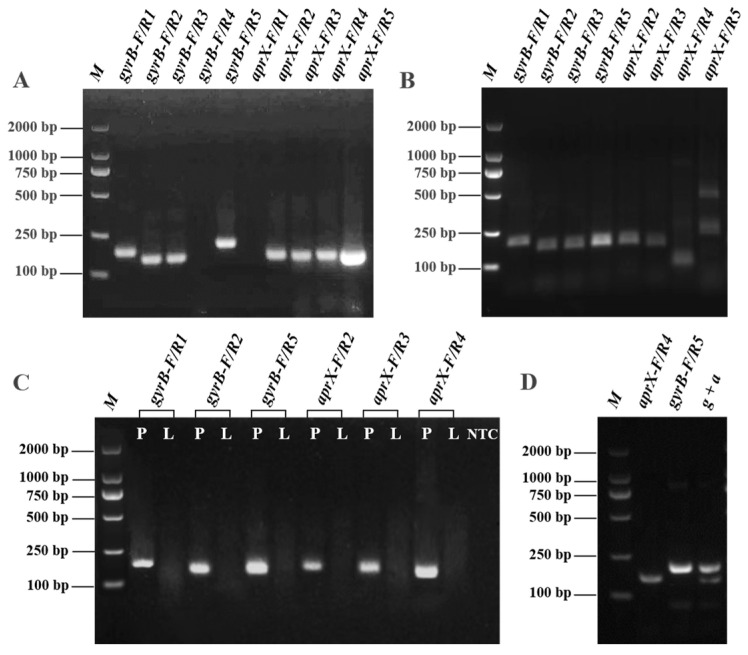
Agarose gel electrophoresis of (**A**) PCR; (**B**) RAA; (**C**) Specific validation of 6 pairs of primers. Note: Each pair of primers was amplified using P (*P. fluorescens*) and non-Pseudomonas L (*L. monocytogenes*) DNA templates, respectively; and (**D**) Cross-reactivity verification between a (*aprX*-F/R4) and g (*gyrB*-F/R5).

Ten pairs of primer information obtained after initial screening by an NCBI-primer-BLAST tool and qPCR assays are presented in Table 1. PCR-AGE and RAA-AGE analyses were conducted to assess primers’ amplification efficiency for *gyrB* and *aprX*. As shown in Figure 2A, it was found that target bands were observed in eight pairs of primers in the PCR-AGE results. Among them, six primer pairs showed single and bright bands between 100 bp and 250 bp (Figure 2B) in RAA-AGE, indicating that the target sequences were successfully amplified.

However, no bands were observed between the primers and non-Pseudomonas *L. monocytogenes*, whereas all primers had distinct bands with *P. fluorescens*, demonstrating that these primers had good interspecies selectivity (Figure 2C). Among these primers, the thickest bands were found in the *gyrB* of the *gyrB*-F/R5 primer pair and the *aprX* of the *aprX*-F/R4 primer pair, respectively, indicating that the amplification effects of these two pairs of primers were the best. Thus, the two pairs of primers were further analyzed by cross-reactivity verification. It was found that when the two primers were simultaneously used for RAA amplification, two clear bands of different molecular weights (*g + a*, Figure 2D) were observed, indicating that there was no interference or cross-reactivity between the two pairs of primers. It ensured the reliability and accuracy of the amplification results of the *gyrB* and *aprX* genes.

### 3.2. Optimization of RAA-TS-DTL Detection System

**Figure 3:**
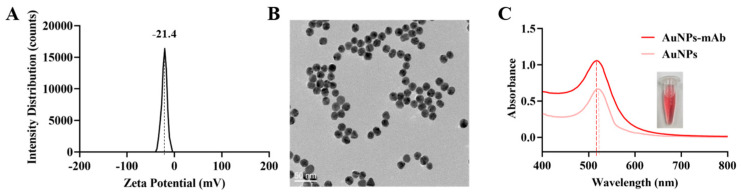
Characterization of AuNPs: (**A**) Zeta potential; (**B**) TEM (**C**) UV-vis of AuNPs and AuNPs probe.

The AuNPs probe was prepared by coating digoxigenin mAb on the surface of the AuNPs. As shown in Figure 3, AuNPs had a surface charge of approximately −21.4 mV (Figure 3A) due to the presence of citrate ions as stabilizing agents [25]. The TEM results indicated that the synthesized AuNPs were predominantly spherical with smooth surfaces and exhibited excellent dispersion (Figure 3B), confirming an average particle size of 21.1 nm. Ultraviolet-visible spectroscopy (UV-Vis) showed that AuNPs had a maximum absorption peak at 520 nm (Figure 3C), which was consistent with the results of Xie et al. [26]. In contrast, a redshift in the absorption peak was found in the AuNPs probe, indicating that the antibody was successfully conjugated onto AuNPs (Figure 3C).

### 3.3. Characterization of AuNPs and Its Probes

**Figure 4:**
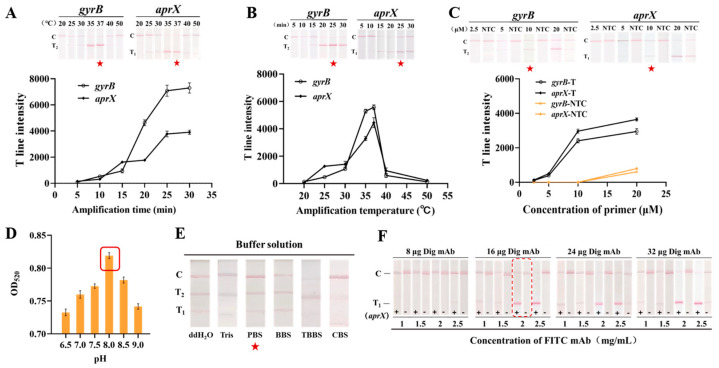
Effects of different conditions of RAA-TS-DTL system. (**A**) RAA reaction temperature, (**B**) RAA reaction time, (**C**) primer concentration, (**D**) solution pH of AuNPs, (**E**) buffer systems, and (**F**) T_1_ line sprayed concentration and probe antibody coupling amount. TBBS: Tween borate-buffered saline solution; CBS: carbonate-buffered saline solution; BBS: borate-buffered saline solution.

To establish an RAA-TS-DTL detection system for *P. fluorescens*, RAA reaction conditions, the pH, and conjugated antibody amounts of the AuNPs probe, a chromatographic buffer, and the coating concentration of the T_1_ line were optimized individually. As shown in Figure 4A, when the RAA-amplified products of *aprX* and *gyrB* at 35 °C and 37 °C were used as samples for TS-DTL detection, obvious T_1_ and T_2_ lines were observed. Quantitative analysis indicated that the intensity of both T lines was the highest at 37 °C. For the amplification time, it was found that the intensity of both T lines tended to be saturated after amplification for 25 min (Figure 4B). In addition, the double T line intensity of *gyrB* and *aprX* increased with the increase in primer concentration and tended to saturate when the concentration of primers was greater than 10 μM. However, a false-positive result was observed for the 20 μM primers (Figure 4C). It was found that excessive primer concentrations might lead to non-specific amplification [27]. Therefore, the optimal RAA amplification condition for *gyrB* and *aprX* was 10 μM primer and reaction at 37 °C for 25 min.

The binding efficiency between the antibody and antigen was influenced by the ionic strength of the chromatographic buffer, the mAb conjugation amount of the probe, and the coating concentration of the T line [23]. It was found that AuNPs probes prepared at pH 8.0 had the highest absorbance value at 520 nm (Figure 4D). Comparing five chromatographic buffers, strong and clear T_1_ and T_2_ lines were observed in the PBS buffer (Figure 4E). Furthermore, 16 or 24 µg digoxigenin mab-coupled probes showed the highest intensity when T_1_ lines were coated with 2 mg/mL FITC (Figure 4F). Considering the detection performance and cost, PBS (pH 7.4) and 16 µg of a digoxigenin mAb-coated AuNPs probe, 2 mg/mL of an FITC antibody-coated T_1_ line were determined as the optimal conditions for TS-DTL detection.

### 3.4. Detection Performance Evaluation of RAA-TS-DTL

#### 3.4.1. Specificity

**Figure 5:**
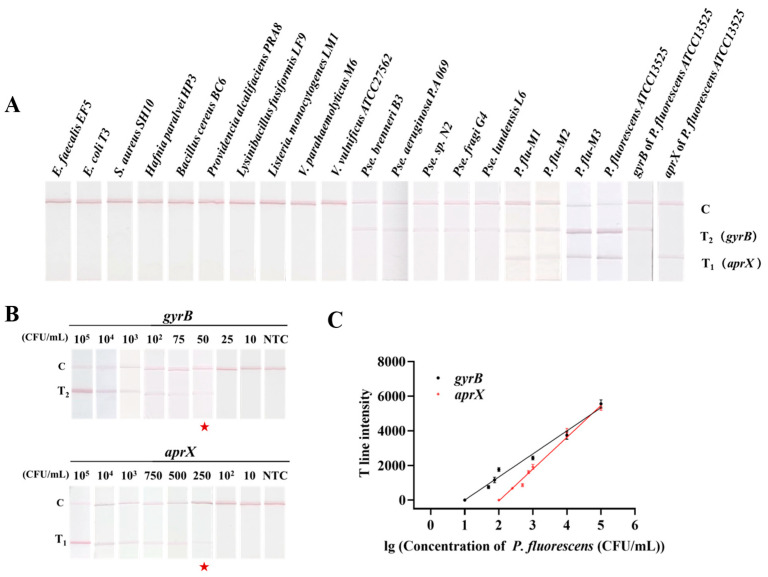
Performance evaluation of RAA-TS-DTL system. (**A**) specificity of 19 strains of bacteria; (**B**) qualitative test results of RAA-TS-DTL single gene T_1_ (*aprX* gene) and T_2_ (*gyrB* gene) and (**C**) quantitative standard curve. NTC: No Template Control.

In this study, nineteen bacterial strains were employed to assess the specificity of the RAA-TS-DTL method, including *P. fluorescens* ATCC 13525, three *P. fluorescens* (M1-3), and five *Pseudomonas* spp. isolated from dairy, and ten common foodborne pathogens such as *E. coli*, *Vibrio* spp., and *Bacillus* spp. (10^5^ CFU/mL, Table S1). As shown in Figure 5A, when employing RAA products amplified specifically from a single *aprX* or *gyrB* gene as the target samples exclusively, the respective T_1_ or T_2_ line was discernible, indicating the absence of cross-reactivity during the individual single gene detection process. Among the five *Pseudomonas* spp. strains tested, solely, the T_2_ line was displayed, whereas in the four strains of *P. fluorescens* (ATCC 13525, M1-3), both T_1_ and T_2_ lines were observed simultaneously, indicating the specificity of the developed RAA-TS-DTL assay for identifying the *P. fluorescens* harboring the *aprX* gene. Furthermore, both T lines were invisible to the other ten species of foodborne pathogens. Therefore, the developed RAA-TS-DTL system was specific for *P. fluorescens*.

#### 3.4.2. Sensitivity

The visual sensitivity of the RAA-TS-DTL assay was evaluated using various concentrations of *P. fluorescens*. As shown in Figure 5B, the intensity of both T lines decreased with the decrease in the *P. fluorescens* concentration. It was evident that the T_1_ signal for *aprX* and the T_2_ signal for *gyrB* disappeared when the concentration of *P. fluorescens* was below 250 CFU/mL and 50 CFU/mL, respectively. Thus, the two concentrations were determined to be the qualitative limit of RAA-TS-DTL for *P. fluorescens*. According to the intensity of dual T lines obtained by Image J software, the quantitative detection curve of the RAA-TS-DTL system is plotted in Figure 5C. The linear fitting formula for the *gyrB* gene was *y* = 1332*x* − 1329 (*R*^2^ = 0.9784), and its quantitative limit of detection (LOD) was calculated to be 37 CFU/mL. While for the *aprX* gene, the linear equation was *y* = 1839*x* − 3734 (*R*^2^ = 0.9844), and its LOD was 233 CFU/mL. Compared to other detection methods for *P. fluorescens* (Table 2), the sensitivity of *aprX* gene was similar with the multiplex qPCR (262 CFU/mL) [19], LAMP (3 × 10^2^ CFU/mL) [28], and RT-LAMP (2.2 × 10^2^ CFU/mL) [20] and greatly higher than that of Duplex PCR (3.5 × 10^3^ CFU/mL) [29]. Therefore, the RAA-TS-DTL method demonstrated exceptional sensitivity.

#### 3.4.3. Accuracy

**Figure 6:**
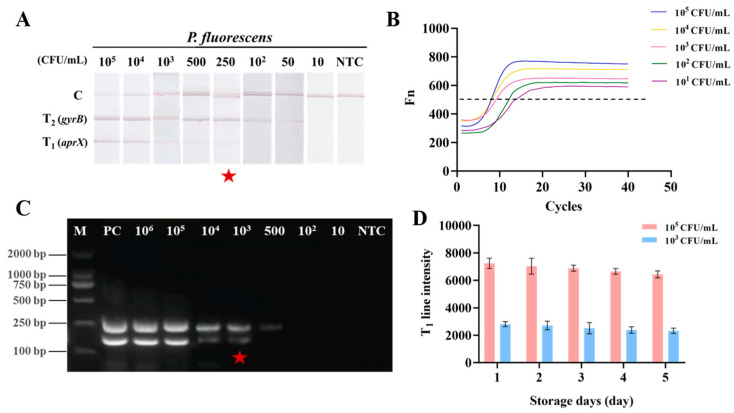
Detection of *P. fluorescens* in UHT milk by (**A**) RAA-TS-DTL, (**B**) qPCR and (**C**) PCR-AGE; (**D**) stability analysis of RAA-TS-DTL for *P. fluorescens*.

To evaluate detection accuracy, UHT milk samples spiked with *P. fluorescens* were used as samples individually for PCR, PCR-AGE, and RAA-TS-DTL detection. Firstly, DNA was extracted from live bacteria and hot-sterilized suspensions of the PMAxx treatment group and control group. PCR-AGE analysis showed that dead bacteria treated with PMAxx lacked amplification bands, and the fluorescence intensity was significantly increased (>0.6), while the fluorescence intensity of other live-bacteria-treated groups was lower than 0.1 (Figure S1). This suggested that PMAxx treatment enhanced the fluorescence of dead bacteria. This is because PMAxx was a dead bacteria DNA modification dye that could cross-link with dead bacteria DNA via the damaged cell membrane, thereby preventing the primer from recognizing and inhibiting its amplification [31]. To ensure accurate and reliable results, we undertook additional optimization efforts targeting the concentration, dark incubation time, and exposure time for bacteria treated with PMAxx. According to the results of PCR-AGE, when the concentration of PMAxx exceeded 20 μM, the dark incubation time surpassed 10 min, the exposure time extended beyond 15 min, and the fluorescence intensity of the sample DNA approached saturation (Figure S2). Therefore, the optimal PMAxx pretreatment conditions were finally determined to be 20 μM PMAxx, followed by 10 min incubation in the dark and a 15 min exposure period. Next, the DNA of different concentrations of *P. fluorescens* after optimal PMAxx treatment was extracted and detected by qPCR, PCR-AGE, and RAA-TS-DTL, respectively. In the developed RAA-TS-DTL system, the T_1_ line corresponding to *aprX* disappeared at a concentration below 250 CFU/mL, while the T_2_ line for *gyrB* disappeared at a lower concentration than 50 CFU/mL of *P. fluorescens* (Figure 6A). Obviously, qPCR showed the best detection sensitivity; the LOD of *aprX* and *gyrB* genes was 10 CFU/mL (Figure 6B). In contrast, dual PCR-AGE bands were discernible at a concentration of 10^3^ CFU/mL of *P. fluorescens* (Figure 6C). This observation showed the qualitative sensitivity of the RAA-TS-DTL system for detecting *P. fluorescens*. Although the detection sensitivity of TS-DTL was lower than that of qPCR, it was fourfold greater compared to the PCR-AGE method. Notably, the established RAA-TS-DTL system could realize the detection of *P. fluorescent* within 90 min, including DNA extraction (30 min) and RAA (25 min), PMAxx pretreatment of the sample (25 min), and TS-DTL detection (10 min). Due to its ease of operation and the absence of the need for specialized equipment such as PCR, the RAA-TS-DTL system is suitable for use as an early and rapid screening or on-site detection method for *P*. *fluorescens*.

#### 3.4.4. Stability

Increasing the temperature to accelerate aging is one of the most common ways to investigate the stability of test strips. To assess the stability, TS-DTL were stored in an oven at 60 °C, and high (10^5^ CFU/mL), medium (10^3^ CFU/mL), and low (10^1^ CFU/mL) levels of *P. fluorescens* were detected daily by the RAA-TS-DTL system for five days. As shown in Figure 6D, little change was found in the intensity of the T_1_ line. For 10^5^ CFU/mL of *P. fluorescens*, the reducing rate was less than 10% on the fifth day, and a similar decrease of 7.8% was found at 10^3^ CFU/mL. For 10 CFU/mL of *P. fluorescens*, neither of the T lines was observed throughout the assay. According to the Arrhenius equation, storing the test strips at 60 °C for one day is equivalent to storing them at 25 °C for 1.2 months. Consequently, the TS-DTL system developed in this study has good stability and may remain stable at 25 °C for about 6 months. However, in the subsequent development of the RAA-TS-DTL kit product, more stability assessment indicators, such as photobleaching, nanoparticle aggregation, and transportation conditions, should be set to test the stability of the reagent and the performance of the product.

#### 3.4.5. Random Testing of Spiked Food Samples

It was possible to evaluate the potential application of the developed RAA-TS-DTL through the analysis of a variety of spiked food samples. A total of 50 different samples, including 10 unspiked samples (negative samples), were prepared, and 40 food samples spiked with *P. fluorescens* following PMAxx treatment were selected randomly for detection by microbial culture, PCR-AGE, and RAA-TS-DTL, respectively. The detection rates of the three methods are shown in Table 3.

The detection rate and accuracy of positive and negative samples by the microbial culture method were 100%. However, PCR-AGE had four false-negative results, and the detection rate of positive samples was 90%, showing a high false-negative rate. For the developed RAA-TS-DTL system, the detection accuracy of *P. fluorescens* in milk by this method was 100%. But there was one false-positive result and one false-negative result, respectively, when it was used to detect the *P. fluorescens* in meat or aquatic products. Dayana et al. found that the fish matrix affected the accuracy of gas chromatography-pulsed flame photometric detection for butyltin compounds [32]. Consequently, the meat matrix probably interfered with the accuracy of RAA-TS-DTL. Furthermore, 40 positive spiked food samples were prepared by adding different concentrations of *P. fluorescent* to milk, meat, and aquatic products. The detection results of the classical microbial culture method and RAA-TS-DTL are shown in Table S2. It was found that the detection results of these two methods for the 40 simulated food samples were all positive, with a consistency of 100%. However, both high and low bacterial concentrations were detected by the two methods, which might have been caused by uneven standard additions or sampling errors.

In summary, the developed RAA-TS-DTL system was simple, rapid, and highly accurate for detecting *P. fluorescens* in milk. It could detect live *P. fluorescens* within 90 min when combined with the sample pretreatment of PMAxx. Due to its ease of operation and the absence of the need for specialized equipment such as PCR, the RAA-TS-DTL system developed in this study is quite suitable for use as an early and rapid screening or on-site detection method for *P*. *fluorescens* in dairy products. At the same time, it can also serve as a new type of food safety monitoring method for food processing enterprises or market supervision departments. However, the RAA-TS-DTL is more suitable for detecting *P. fluorescens* in milk. In the future, it is necessary to study how to eliminate the interfering effects of food substrates such as aquatic products.

## 4. Conclusions

In this study, based on the virulence gene *aprX* of *P. fluorescens* and the housekeeping gene *gyrB* of *Pseudomonas*, a rapid, sensitive, and accurate RAA-TS-DTL system was developed for the detection of *P. fluorescens* in milk. Combined with the sample pretreatment of PMAxx, the simple detection of *gyrB* (50 CFU/mL) and *aprX* (250 CFU/mL) in live *P. fluorescens* was realized through naked-eye observation. In addition, this method was specific and highly sensitive for both *gyrB* and *aprX* genes of *P. fluorescens*; their LODs were four times that of the PCR-AGE method. The RAA-TS-DTL system could accurately detect *P. fluorescens* spiked in milk, and the consistency with traditional culture methods reached 100%. Furthermore, the entire testing process (including sample pretreatment and RAA) could be completed within 90 min. Therefore, it is not only reliable but also provides technical support for the rapid screening and on-site detection of pathogenic *P. fluorescens* in foods.

## Figures and Tables

**Table 1:** Sequences of the Primers Set for *P. fluorescens*.

Assay	Name	Sequence (5′-3′)	Amplicon Size (bp)
RAA	*aprX*-F1	CTTACTCGCAAATCGATAGCTTCAGCCATT	180
	*aprX*-R1	CCGAGGTGAGGAAGGTGTAGGTCAGATC	
	*aprX*-F2	TCAACCTCACTGCCACCTCGTTCTCTGATG	143
	*aprX*-R2	CTTGATGGTGTTGGCGACTTGGTTACCAATGAT	
	*aprX*-F3	GGGATGGTGCTGCGTACCGGGACTTTGATG	151
	*aprX*-R3	AGTGCGGCCTGTGCTTTCTGCTGGGTGTTG	
	*aprX*-F4	TGACCAACAACAGCTACACGCCGAACAAGA	157
	*aprX*-R4	CCATAGGTGGCGTCGTTATAGGTCGGGTTG	
	*aprX*-F5	GGATGGTGCTGCGTACCGGGACTTTGATGG	150
	*aprX*-R5	AGTGCGGCCTGTGCTTTCTGCTGGGTGTTG	
TS- DTL	*aprX*-F4-FAM	FAM-TGACCAACAACAGCTACACGCCGAACAAGA	157
	*aprX*-R4-Dig	Digoxigenin-CCATAGGTGGCGTCGTTATAGGTCGGGTTG	
RAA	*gyrB*-F1	CGCGTAAAGCCCGTGAGATGACCCGCCGTAA	141
	*gyrB*-R1	CGGAACCGCCAGCAGAGTCACCTTCCACCAA	
	*gyrB*-F2	GGCGGTCTGCGTGCGTTCGTTGAATACCTGA	118
	*gyrB*-R2	TCCACTGCAAAGCGATTTCTACGCCGATGCC	
	*gyrB*-F3	CCGTACCCTGCTGCTGACCTTCTTCTTCCG	128
	*gyrB*-R3	CGTCTTTGATGTATTGCTCTTGCTTGCCCTTT	
	*gyrB* F4	AACATCGACAAGCTGCGCTACCACAACATCA	97
	*gyrB*-R4	GGAAGAAGAAGGTCAGCAGCAGGGTACGGAT	
	*gyrB*-F5	GCCAAGCGTATTCGTGAGCTGTCTTTCCTTA	207
	*gyrB*-R5	CAAAGCGATTTCTACGCCGATGCCATCTTCA	
TS- DTL	*gyrB*-F5-Bio	Biotin-CCGTACCCTGCTGCTGACCTTCTTCTTCCG	207
	*gyrB*-R5-Dig	Digoxigenin-CGTCTTTGATGTATTGCTCTTGCTTGCCCTTT	

**Table 2:** Comparison of different detection methods for *P. fluorescens*.

Method	Target Gene	LOD	Test Time	Reference
Multiplex qPCR	*aprX*	262 CFU/mL	3 h	[19]
Multiplex PCR	*gyrB*	9 CFU/mL	3.5 h	[30]
Duplex PCR	*aprX*	3.5 × 10^3^ CFU/mL	3.5 h	[29]
LAMP	*aprX*	3 × 10^2^ CFU/ mL	20 min	[28]
RT-LAMP	*gyrB*/*aprX*	2.2 × 10^2^ CFU/ mL	20 min	[20]
RAA-TS-DTL	*gyrB* *aprX*	37 CFU/ mL 233 CFU/ mL	35 min	This study

**Table 3:** Accuracy results of RAA-TS-DTL based on random testing.

Sample Type	Number of Spiked Samples	Number of Unspiked Samples	Number of Samples Detected (+/−)					
			Microbial Culture		PCR-AGE		This Study	
			+	−	+	−	+	−
Dairy	20	5	20	5	19	6	20	5
Meats	12	3	12	3	10	5	13	2
Aquatic products	8	2	8	2	7	3	9	1
Total	40	10	40	10	36	14	42	8

(Note: +, positive result; −, negative result).

## Data Availability

The original contributions presented in this study are included in the article. Further inquiries can be directed to the corresponding author(s).

## Supplementary Materials

The following supporting information can be downloaded at: https://www.mdpi.com/article/10.3390/bios15080553/s1, Figure S1: Effect of PMAxx on live and dead bacteria. (A) PCR-AGE; (B) Fluorescence intensity. NTC: No Template Control.; Figure S2: Optimization of PMAxx concentration (A), dark incubation time (B), and exposure time (C) on fluorescence intensity and PCR-AGE of sample bacteria. BC: No PMAxx treatment, NTC: No Template Control.; Table S1: Bacteria strains used in this study.; Table S2: The *P. fluorescens* in different foods detected by RAA-TS-DTL and microbial culture methods.
