# Supplementary material for: A Novel Sensitive Recombinase-Aided Amplification Integrated Test Strip for Pseudomonas fluorescens in Milk via Dual Gene Probes
Source: Biosensors (Basel). 2025 Aug 21;15(8):553. doi: 10.3390/bios15080553 (PMC12385110; doi:10.3390/bios15080553)
Supplement: Supplementary file 1 [file biosensors-15-00553-s001.zip › biosensors-3771872-supplementary.pdf]

biosensors

Supporting information

A Novel Sensitive Recombinase-Aided Amplification Integrated Test Strip for *Pseudomonas Fluorescens* in Milk via Dual Gene Probes

Guangying Zhang [1,2,†], Lili Zhang [1,2,†], Jingqin Ye [3], Dongshu Wang [3,*] and Ying Lu [1,2,4,*]

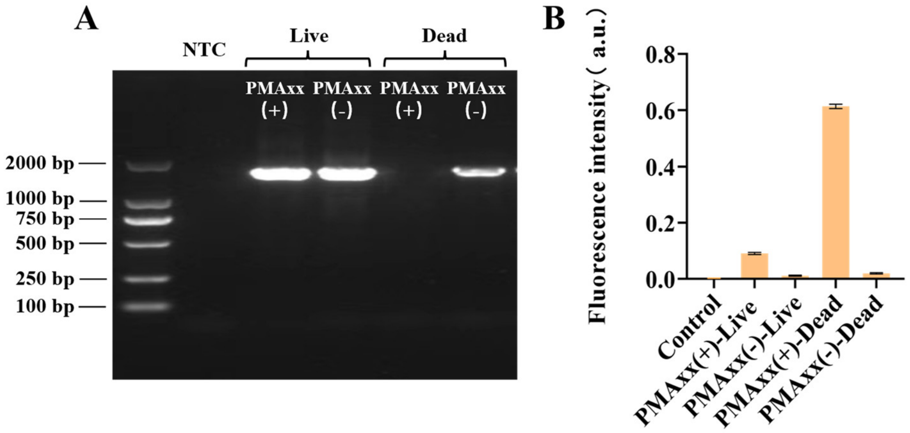

Figure S1. Effect of PMAxx on live and dead bacteria. (A) PCR-AGE; (B) Fluorescence intensity. NTC: No Template Control.

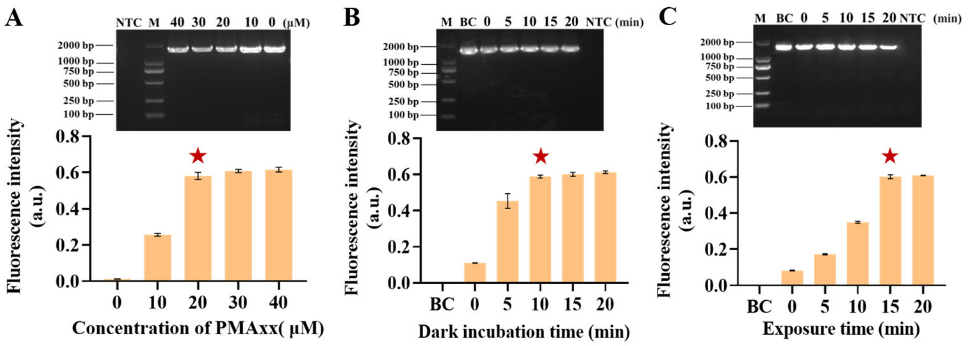

Figure S2. Optimization of PMAxx concentration(A), dark incubation time (B), and.exposure time (C) on fluorescence intensity and PCR-AGE of sample bacteria. BC: No PMAxx treatment, NTC: No Template Control.

Table S1. Bacterial Strains Used in this Study.

Source	Bacterial strains	Species and Strain
Foodborne strain	*Pseudomonas sp.*	*Pse. sp.* N2
	Pseudomonas brenneri	*Pse. brenneri* B3
	Pseudomonas fragi	*Pse. fragi* G4
	Pseudomonas lundensis	*Pse. lundensis* L6
	Pseudomonas fluorescens	*P. flu* M1-7
	Pseudomonas aeruginosa	*P.A* 069
	Listeria. monocytogenes	*L. monocytogenes* LM1
	Lysinibacillus fusiformis	*L. fusiformis* LF9
	Providencia alcalifaciens	*Pro. alcalifaciens* PRA8
Environmental strain	*V. parahaemolyticus*	*V. p* M6
	Bacillus cereus	*B. cereus* BC6
	Hafnia.paralvei	*H. paralvei* HP3
	Staphylococcus aureus	*S. aureus* SH10
	Enterococcus coli	*E. coli* T3
	Enterococcus faecalis	*E. faecalis* EF5
Reference strain	*Pseudomonas fluorescens*	*P. fluorescens* ATCC13525
	Vibrio vulnificus	*V. vulnificus* ATCC27562

Table S2. The *P. fluorescens* in different foods detected by RAA-TS-DTL and microbial culture methods.

Food type	Sample number	Spiked concentration of *P. fluorescens* (CFU/mL)	Detected *P. fluorescens* (CFU/mL)		
			Microbial culture	RAA-TS-DTL	Result determination
Dairy	1	5.0×10^{3}	6.6×10^{3}	2.1×10^{3}	Positive
	2	5.0×10^{3}	8.9×10^{3}	5.8×10^{3}	Positive
	3	5.0×10^{3}	1.1×10^{4}	7.7×10^{3}	Positive
	4	5.0×10^{3}	5.4×10^{3}	3.4×10^{3}	Positive
	5	5.0×10^{3}	7.8×10^{3}	6.0×10^{3}	Positive
	6	5.0×10^{4}	5.2×10^{4}	2.4×10^{4}	Positive
	7	5.0×10^{4}	3.7×10^{4}	3.3×10^{4}	Positive
	8	5.0×10^{4}	4.1×10^{4}	2.7×10^{4}	Positive
	9	5.0×10^{4}	2.5×10^{4}	9.8×10^{3}	Positive
	10	5.0×10^{4}	4.1×10^{4}	3.8×10^{4}	Positive
	11	5.0×10^{5}	4.3×10^{5}	3.3×10^{5}	Positive
	12	5.0×10^{5}	9.4×10^{4}	1.9×10^{5}	Positive
	13	5.0×10^{5}	6.9×10^{5}	5.5×10^{5}	Positive
	14	5.0×10^{5}	3.2×10^{5}	1.2×10^{5}	Positive
	15	5.0×10^{5}	4.8×10^{5}	6.8×10^{5}	Positive
	16	5.0×10^{6}	1.5×10^{7}	5.2×10^{6}	Positive
	17	5.0×10^{6}	5.6×10^{6}	4.7×10^{6}	Positive
	18	5.0×10^{6}	8.7×10^{6}	4.8×10^{6}	Positive
	19	5.0×10^{6}	7.3×10^{6}	3.5×10^{6}	Positive
	20	5.0×10^{6}	6.2×10^{6}	2.9×10^{6}	Positive
Meats	21	5.0×10^{3}	2.7×10^{3}	1.3×10^{3}	Positive
	22	5.0×10^{3}	6.1×10^{3}	4.5×10^{3}	Positive
	23	5.0×10^{3}	9.8×10^{3}	8.6×10^{3}	Positive
	24	5.0×10^{4}	3.9×10^{4}	8.1×10^{3}	Positive
	25	5.0×10^{4}	5.7×10^{4}	4.9×10^{4}	Positive
	26	5.0×10^{4}	2.1×10^{4}	8.8×10^{3}	Positive
	27	5.0×10^{5}	4.4×10^{5}	4.1×10^{5}	Positive
	28	5.0×10^{5}	7.9×10^{5}	8.7×10^{5}	Positive
	29	5.0×10^{5}	8.8×10^{5}	9.3×10^{5}	Positive
	30	5.0×10^{6}	1.3×10^{6}	2.3×10^{6}	Positive
	31	5.0×10^{6}	7.4×10^{6}	3.9×10^{6}	Positive
	32	5.0×10^{6}	2.8×10^{6}	1.6×10^{6}	Positive
Aquatic products	33	5.0×10^{3}	4.4×10^{3}	4.7×10^{3}	Positive
	34	5.0×10^{3}	7.5×10^{3}	4.9×10^{3}	Positive
	35	5.0×10^{4}	1.6×10^{4}	3.2×10^{4}	Positive
	36	5.0×10^{4}	8.3×10^{4}	1.3×10^{5}	Positive
	37	5.0×10^{5}	4.2×10^{5}	2.6×10^{5}	Positive
	38	5.0×10^{5}	3.5×10^{5}	7.4×10^{5}	Positive
	39	5.0×10^{6}	5.1×10^{6}	1.1×10^{6}	Positive
	40	5.0×10^{6}	9.6×10^{6}	5.4×10^{6}	Positive
